# Discrete Event Simulation for Decision Modeling in Health Care: Lessons from Abdominal Aortic Aneurysm Screening

**DOI:** 10.1177/0272989X17753380

**Published:** 2018-04-02

**Authors:** Matthew J. Glover, Edmund Jones, Katya L. Masconi, Michael J. Sweeting, Simon G. Thompson, SWAN Collaborators, Janet T. Powell

**Affiliations:** Health Economics Research Group, Brunel University London, Uxbridge, Middlesex, UK; Cardiovascular Epidemiology Unit, Department of Public Health and Primary Care, University of Cambridge, Cambridge, Cambridgeshire, UK; Cardiovascular Epidemiology Unit, Department of Public Health and Primary Care, University of Cambridge, Cambridge, Cambridgeshire, UK; Cardiovascular Epidemiology Unit, Department of Public Health and Primary Care, University of Cambridge, Cambridge, Cambridgeshire, UK; Cardiovascular Epidemiology Unit, Department of Public Health and Primary Care, University of Cambridge, Cambridge, Cambridgeshire, UK

**Keywords:** abdominal aortic aneurysm, decision analytic model, discrete event simulation, Markov model, screening

## Abstract

Markov models are often used to evaluate the cost-effectiveness of new healthcare interventions but they are sometimes not flexible enough to allow accurate modeling or investigation of alternative scenarios and policies. A Markov model previously demonstrated that a one-off invitation to screening for abdominal aortic aneurysm (AAA) for men aged 65 y in the UK and subsequent follow-up of identified AAAs was likely to be highly cost-effective at thresholds commonly adopted in the UK (£20,000 to £30,000 per quality adjusted life-year). However, new evidence has emerged and the decision problem has evolved to include exploration of the circumstances under which AAA screening may be cost-effective, which the Markov model is not easily able to address. A new model to handle this more complex decision problem was needed, and the case of AAA screening thus provides an illustration of the relative merits of Markov models and discrete event simulation (DES) models. An individual-level DES model was built using the R programming language to reflect possible events and pathways of individuals invited to screening v. those not invited. The model was validated against key events and cost-effectiveness, as observed in a large, randomized trial. Different screening protocol scenarios were investigated to demonstrate the flexibility of the DES. The case of AAA screening highlights the benefits of DES, particularly in the context of screening studies.

Modeling will almost always constitute an essential component of an economic evaluation to inform decision making, to overcome the limitations of available randomized trial data.^1^ In screening studies, where much of the cost is upfront and benefits accrue over a long period of time, there is a need for modeling approaches that can contribute to long-term economic evaluations. The choice of modeling technique is at the discretion of the analyst and often reflects an implied trade-off between simplicity and realism in reflecting a disease’s natural history, treatment, and patient outcomes.^2,3^ Markov models have been widely used, as they provide a simple mechanism to estimate the long-term costs and effects of healthcare interventions.^4^ Discrete event simulation (DES) is a less common alternative that avoids the use of states and fixed cycle lengths and instead models events at the individual level. Some have suggested that DES should always be preferred, whereas others have highlighted particular circumstances where DES should be favoured.^5,6^ The case of abdominal aortic aneurysm (AAA) screening offers an illustration of the relative merits of these techniques.

An AAA is commonly defined as an aortic diameter ≥3.0 cm. A long-term Markov model demonstrated that offering population screening for AAA to men aged 65 y in the UK was likely to be highly cost-effective.^7^ This model was largely populated using data from the 4-y follow-up of the randomized Multicentre Aneurysm Screening Study (MASS),^8^ and adopted the same screening methods, surveillance intervals (for 3.0 to 5.4 cm AAAs) and AAA diameter threshold (5.5 cm) for referral for elective surgical intervention as in MASS. The MASS trial of 67,800 men aged 65 to 74 showed that an invitation to a one-off ultrasound scan and surveillance or referral for elective surgical intervention of identified AAAs was effective in reducing AAA-related mortality, initially at the 4-y follow-up,^8^ and subsequently at longer-term follow-up.^9^ The results from the modeling and the MASS trial formed a large part of the evidence base supporting the phased implementation from 2009 of the NHS AAA Screening Programme (NAAASP) in England, with full coverage across the UK by the end of 2013.

Research into the clinical and cost-effectiveness of AAA screening has evolved since the first Markov modeling was performed, with the emergence of new data and evidence. Initial observational data from NAAASP suggested that the current prevalence of AAAs is substantially lower than that observed in MASS (1.6% v. 4.9%). The Markov model (MM) was updated to reflect this lower prevalence as well as changes in costs, the increased use of endovascular surgical techniques, meta-analyzed data on growth and rupture rates,^10,11,11^ and longer-term MASS follow-up.^12^ The results suggested screening is still likely to be highly cost-effective, with a long-term incremental cost-effectiveness ratio of £7,370 (95%CI, £5,467 to £9,443) per quality-adjusted life year (QALY). Other studies based on populations in Denmark^13^ and Sweden,^14^ with similar AAA prevalence, support this conclusion.

However, different programs and randomized trials have adopted diverse surveillance intervals, with little consensus on optimal intervals.^15^ More substantial surveillance data from the RESCAN project was incorporated into an adapted MM to investigate different surveillance intervals, and the results suggested that lengthening the time between rescans for men with the smallest aneurysms could be done at acceptable clinical risk^10^ and would be a cost-effective strategy.^11^ Some of the protocols around screening have also come under scrutiny. For example, the definition of an AAA as an aortic diameter ≥3.0 cm is somewhat arbitrary: there is evidence that many individuals with screen-detectable sub-aneurysmal aortic dilation (2.5 to 2.9 cm) will progress to AAA within 10 y.^16^ The implications for screening remain unclear. There have also been some suggestions that the surgical threshold itself should be altered.^17^

In light of these findings, the decision problem no longer relates only to “screening” v. “no screening” for older men but has evolved to include the circumstances under which screening may be cost-effective.^18-21^ Modeling allows these questions to be addressed without conducting costly primary research and could extend to varying a number of fixed parameters (e.g., surveillance intervals, the AAA diameter threshold for referral for elective surgery, screening of women, targeted screening based on patient characteristics). However, MMs can be inflexible. This inflexibility was demonstrated by the extensive re-programming needed to build tunnel states in the MM when the model was adapted to assess different surveillance intervals.^11^ Such analyses are important for existing programs aiming to improve their performance or extend population coverage, as well as for other countries considering implementation. Therefore, a model better able to handle this decision problem is required. This paper describes: 1) the development and validation of a DES model to estimate the clinical and cost-effectiveness of AAA screening; and 2) the use of the DES to explore the cost-effectiveness of screening under various scenarios, which was not possible with the original MM.

## Methods

### Development of a Simulation Model

A DES was implemented using the freely available statistical programming language R and based on the original MM.^7^ The original MM defined several health states that related to AAA identification, aortic diameter (<3.0 cm, 3.0 to 4.4 cm, 4.5 to 5.4 cm, ≥5.5 cm) and associated events (rupture, surgical consultations, elective and emergency AAA repair, death). A set of transition probabilities determined movements between health states for the two populations (invited to screening and not invited). The MM operates at the cohort level: events, mean costs, and QALYs are calculated from the proportions of the cohort that inhabit the different health states in each 3-mo cycle. In contrast, the DES functions at an individual level, simulating sequences of events that occur as a continuous process over time and calculating the associated mean costs and QALYs. It allows individual patient heterogeneity to be characterized and accounts for events as they occur, removing the need for any assumptions relating to averaging costs or outcomes across cycles.

### An Event Scheduling Approach

**Figure 1:**
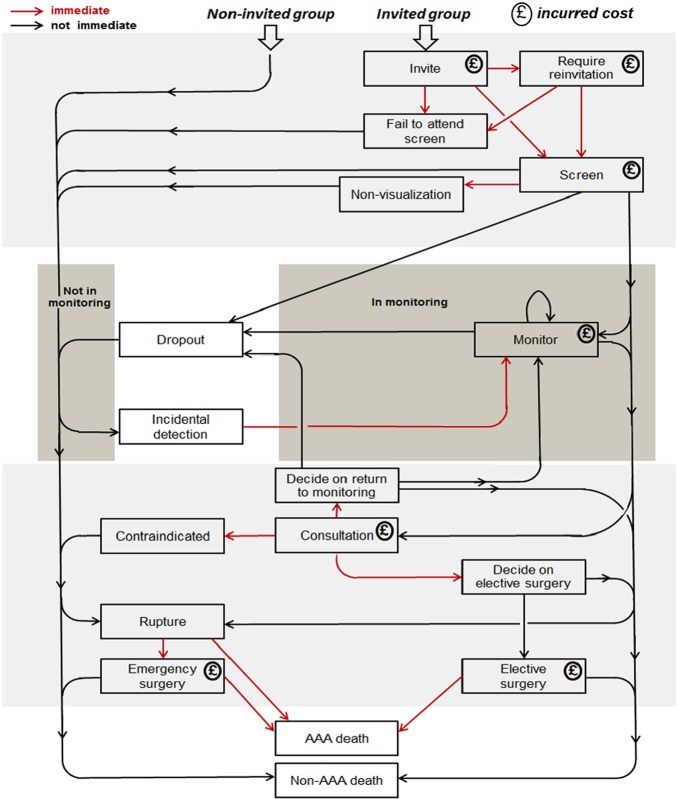
Possible sequences of events in the abdominal aortic aneurysm (AAA) screening discrete event simulation model.

Full details of the DES are available in the SWAN project National Institute for Health Research Health Technology Assessment monograph. The DES adopts an event-scheduling approach by generating a sequence of events for each individual, using a list of events that are “scheduled” for the future (future events list; FEL). The DES has an explicit simulation clock, chooses the event that has the earliest sampled time, and records it in the individual’s sequence of events. It then schedules, reschedules, or cancels other scheduled events as necessary, updating the FEL (for example, if a surveillance rescan finds that an individual’s aortic diameter is above the threshold for elective surgery, then a consultation is scheduled). This process is repeated until death or censoring (dependent on model time horizon). The possible sequences of events are shown in Figure 1.

Individuals are assigned an aortic diameter, drawn from a population distribution, and a latent parameter describing the growth rate of their aorta over time. Details of the aortic growth model are given in the Supplementary Material. Non-AAA death and AAA rupture events are scheduled in the future, and if the individual is in the “invited” group, then an invitation is also scheduled. If the individual is in the “non-invited” group, then an “incidental detection” event is scheduled. The time to AAA rupture is dependent on the individual’s initial aortic diameter and their latent growth rate. In most instances, the scheduled AAA rupture time will be so far in the future that there is no chance of the event occurring. The “incidental detection” event is scheduled to occur only after the time at which an individual’s aortic diameter reaches the diagnosis threshold (e.g., 3.0 cm).

The DES simulates people in pairs, like identical twins, one of whom is in the invited group and one in the non-invited group. The twins have certain characteristics in common: they have the same times of non-AAA death and AAA rupture in their FELs, the same initial aortic diameter and growth rate, and the same values of certain parameters, such as indicators (binary variables) for whether they would be contraindicated for surgery and whether they would survive emergency surgery. Differences in costs and outcomes result from the different events that are scheduled due to involvement or otherwise in the screening programme.

### Joint Continuous AAA Growth and Rupture Model

A major difference between the DES and the MM is that the DES uses a joint continuous-time model for aortic growth and rupture^11^ rather than defining 4 AAA size states. Additionally, when an individual’s aorta is scanned, the measurement is generated by calculating the diameter according to this model and adding measurement error, which is specific to the type of scan used (i.e., ultrasound or computed tomography [CT]). Further alterations related to the move from fixed cycles to a continuous process were made. For example, surgical waiting time was previously separated into two periods: the time from discovery to consultation (71 d) and from consultation to surgery (59 d).^22^ In the MM, it was assumed that this total waiting time could be considered as a 3-mo cycle. The DES would enable these periods to be easily changed if appropriate.

### A Hierarchy of Functions

**Figure 2:**
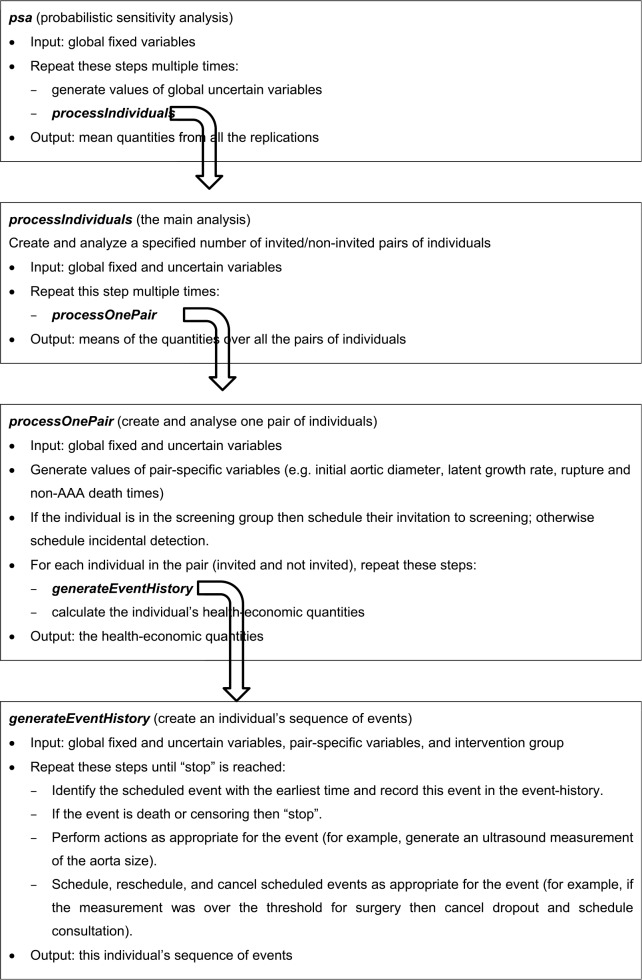
Abdominal aortic aneurysm (AAA) screening discrete event simulation model: Hierarchy of functions.

The R program for the DES is made up of a hierarchy of functions or routines: 1) a probabilistic sensitivity analysis (PSA), which consists of running the main analysis multiple times; 2) the main individual patient simulation analysis, which consists of simulating and analyzing multiple pairs of individuals; 3) the function to process one pair; and 4) the function to generate a sequence of events for an individual. These functions are shown in Figure 2.

The R code developed for this project is available on request from the authors.

The DES involves a large number of parameters. These can be classified into several sets: global fixed parameters, global uncertain parameters, and parameters that are specific to an individual or a pair of twins (“global” refers to population parameters and “uncertain” means that a parameter follows a random distribution). Like the functions, these sets form a hierarchy. For example, in a PSA, a beta distribution is used to generate the probability that an individual will die following emergency surgery, if they have emergency surgery. The parameters of the beta distribution are global fixed parameters, and the probability is a global uncertain parameter. In the main analysis, when a pair of twins is created, the probability is used as the parameter in a Bernoulli distribution to generate the indicator for the twins’ emergency surgery outcomes. The indicator is a variable specific to the pair of twins.

The hierarchy extends downward by two more levels. When the DES generates an individual’s sequence of events, it needs to record the intervention group (i.e., invited or not), which can be regarded as an individual-specific parameter, and when an aorta measurement is generated, a new and unique value of the measurement error is created, which can be regarded as an event-specific parameter. Figure 2 shows how the sets of parameters are passed from one function to another. The definitions of the parameters require judgement and depend on the nature of the input data. For instance, the costs of the scans and other events could be defined as global fixed parameters, if their values are known with great certainty, or global uncertain parameters if they are not.

### Cost-effectiveness Analysis

The cost-effectiveness analysis consists of simulating a large number of individuals, calculating their life-years and costs, and calculating the mean life-years and costs over all the patients in the groups invited to screening and not invited. Given that the model outputs are driven by those individuals who have an AAA, these individuals were oversampled (and later calculations were adjusted to account for this), which reduces considerably the Monte Carlo error when estimating incremental effects and costs. PSA is conducted to account for uncertainty in the model parameters; repeated sets of values for the global uncertain parameters are generated, and the main analysis is run for each set of values. The mean incremental cost and effectiveness (i.e., QALYs), together with the incremental cost-effectiveness ratio (ICER) and incremental net monetary benefit (INMB), are calculated for each set, and the distribution of values is used to estimate the probability that the screening program is cost-effective.

### Programming Practice

The DES is a moderately complicated computer program, and it was therefore necessary to follow basic principles of good programming to ensure that it would run correctly and be maintainable and usable in the future. For example, each function has a clearly defined single purpose that can easily be understood from its name, and priority was given to making the source-code simple, clear, and readable (by other people) rather than computationally fast. The DES was written using a mostly “functional” programming style: the basic building-blocks are functions, and functions do not modify things outside themselves but simply perform actions and then either display output or return relevant quantities (e.g., parameters, event-times). R is convenient for statistical and scientific programming, and it allows loops (or iterative processes) to be written to run in parallel (which is not the case with all programming languages). The DES is eminently suited to parallelization, which speeds it up considerably.

### Model Validation

The original MM was validated against the MASS trial 4-y follow-up to check the appropriateness of model outputs. The validation involved comparing the numbers of key events (e.g., AAA ruptures, number of elective operations) and mean costs and life-years, as observed in the trial, with model outputs based on a simulated population of the same size. The MM was able to replicate the observed data reasonably.^7,23^ For the DES, a similar process was carried out, again using the 4-y MASS follow-up data. Costs and life-years were discounted at 6% and 1.5% per y, respectively, to be consistent with the original rates used in the 4-y follow-up analysis.

Input parameters for the DES were derived from the MASS 4-y follow-up, where possible, including non-AAA death rates, to enable validation. Other adaptations were made to improve the DES model fit to the observed MASS outputs. The parameters for the aorta growth model were chosen such that, at baseline, there were the same proportions of individuals with aortic size <3.0 cm, 3.0 to 4.4 cm, 4.5 to 5.4 cm and ≥5.5 cm as in MASS. Growth rates were based directly on those observed in the screen-detected MASS population, with growth rates for those 2.0 to 2.9 cm extrapolated from a fitted mixed model, and growth rates set to zero for those <2.0 cm at baseline (see Supplementary Material for more details). All aorta measurements that were performed by CT scan (at consultation only) were, on average, 0.24 cm larger than an ultrasound scan, to account for CT scanning measuring outer-to-outer rather than inner-to-inner diameters.^11^ Individuals were censored at uniformly random times between 3 and 5.25 y, because the “4-y” follow-up of the MASS data had censoring times similar to this uniform distribution. Full details of the input parameters and characterization of uncertainty is detailed in Supplementary Table 1.

### New Model Scenarios

After validation against the MASS trial 4-y follow-up, parameter values in the DES were updated to reflect more contemporaneous estimates, the full details of which are provided in Supplementary Table 2. National mortality statistics^24^ were used for non-AAA death rates. The NAAASP baseline aortic diameter distribution was used in the aorta growth and rupture model, with growth and rupture rates based on RESCAN data.^25^ As before, growth rates <3.0 cm were extrapolated from a model or were set to zero. Costs, attendance rate, and other parameters were updated as described by Glover and others,^12^ with QALYs estimated by applying population norm utility weights to life-years accrued. The model structure was further altered to allow for endovascular aneurysm repair (EVAR) as well as open repair, with a proportion of surgery by EVAR, which incurred a different cost and post-operative mortality rate compared to open surgery.^11^ The base case was run over a 30-y time horizon for 65-y-old men invited or not invited to screening, for 10 million pairs of individuals. PSA, based on 1,000 runs with 500,000 pairs of individuals, was used to characterize uncertainty in input parameters. Costs and QALYs were discounted at 3.5% per y.

Two modeling scenarios were explored, showing the flexibility of the DES to estimate the cost-effectiveness of screening under various protocols. The first built on an analysis previously performed using the MM, to identify more cost-effective surveillance intervals for men in the screening program.^11^ The second allowed the inclusion of surveillance for men with sub-aneurysmal aortic diameters (2.5 to 2.9 cm at first screen). Each of these different scenarios was compared to the existing program in terms of costs and QALYs.

#### Scenario 1: Different Surveillance Intervals

Analysis performed using the MM suggested that lengthening surveillance intervals for the smallest identified AAAs would be cost-effective according to thresholds commonly adopted in the UK (£20,000 to £30,000 per QALY).^11^ However, the different surveillance strategies considered were limited to varying the time between monitoring for two AAA size groups (3.0 to 4.4 cm, and 4.5 to 5.4 cm). Unlike the MM, the DES can be easily adapted to use any number of plausible AAA size cutoffs, or to consider differing surveillance intervals. Here, the current NAAASP surveillance strategy of 1-year (3.0 to 4.4 cm AAAs) and 3-month (4.5 to 5.4 cm AAAs) intervals is compared to a strategy of 2-year (3.0 to 3.9 cm AAAs), 1-year (4.0 to 4.4 cm AAAs), and 3-month (4.5 to 5.4 cm AAAs) intervals.

#### Scenario 2: Inclusion of Sub-aneurysmal (2.5 to 2.9 cm) Aortas

The threshold definition of an AAA used in the DES was lowered from 3.0 cm to 2.5 cm. Individuals identified with an aortic diameter of 2.5 to 2.9 cm had surveillance scans scheduled at intervals of 5 years, with the intervals currently adopted by NAAASP maintained for AAAs between 3.0 cm and 5.4 cm.

## Results

### DES Model Validation

**Figure 3:**
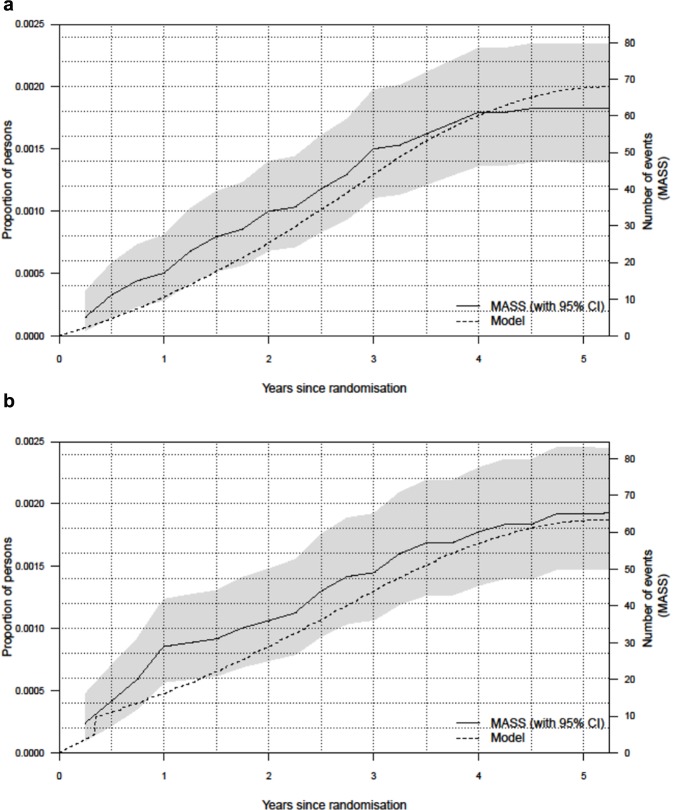
Cumulative numbers of events in the 4-y MASS data and the DES for: (a) emergency operations in the non-invited group and (b) AAA deaths in the invited group. AAA, abdominal aortic aneurysm; DES, discrete event simulation; MASS, Multicentre Aneurysm Screening Study.

The DES validated reasonably against the MASS 4-y data. The DES broadly agreed with both the observed MASS 4-y follow-up and the original MM in terms of differences in life-years and costs (Table 1). However, like the MM, the 4-y ICER for the DES was higher than in the MASS data. Table 2 shows the total numbers of events as observed in MASS and estimated by the original MM and the DES. For most events, the numbers of events were similar, as were the ratios of events in the DES to events in MASS show. The numbers of non-AAA deaths matched very closely, primarily because the MM and DES used non-AAA death rates from MASS and most individuals do not experience AAA rupture. As examples of cumulative events over time, Figure 3 shows the numbers of emergency operations in the non-invited group and AAA deaths in the invited group estimated by the DES, compared to the observed numbers in the MASS 4-y follow-up.

**Table 1:** Life-years and Costs According to the 4-y MASS Follow-up, Markov Model (Kim and others^7^) and the DES^a^

	MASS Observed	Markov Model	DES Model
Non-invited group			
Life-years	3.816	3.905	3.753
Cost	£35.03	£32.74	£39.11
Invited group			
Life-years	3.819	3.907	3.754
Cost	£98.42	£98.32	£101.97
Difference			
Life-years	0.0022	0.0017	0.0015
Cost	£63.39	£65.58	£62.86
ICER	£28,400	£37,700	£42,137
(95% CI)	(£15,000, £146,000)	(£19,700, £147,000)	(£19,935, £3,277,596)^b^

DES, discrete event simulation; ICER, incremental cost-effectiveness ratio; MASS, Multicentre Aneurysm Screening Study.

a Life-years discounted at 1.5% per y and costs at 6% per y.

b Reported as uncertainty interval produced by 1,000 probabilistic sensitivity analysis (PSA) iterations (after assigning ICERs with negative incremental effects and positive costs to be infinite). Mean estimates from 1,000 PSA iterations for the difference in life-years, costs, and the ICER were 0.0015, £62.91 and £46,032, respectively.

**Table 2:** Key Events Observed in the MASS 4-y Follow-up, and as Estimated by the Markov Model (Kim and others^7^) and the DES

	MASS Observed	Markov Model^a^	DES Model^a^	DES Model (% of MASS)
No invitation group				
Elective operation	100	83	98	98
Emergency operation	62	62	68	110
Rupture	138	141	154	112
Contraindicated for elective surgery	NA	14	16	NA
AAA death	113	109	120	106
Non-AAA death	3,750	3,724	3,696	99
Invited group				
Elective operation				
Resulting from screen detection	295	282	330	112
Resulting from incidental detection	31	25	27	86
Emergency operation	28	34	30	106
Rupture	66	78	67	102
Contraindicated for elective surgery				
Resulting from screen detection	41	46	54	131
Resulting for incidental detection	NA	5	5	NA
AAA death	65	69	63	98
Non-AAA death	3,694	3,724	3,700	100
Loss to recall follow-up	290	289	278	96

AAA, abdominal aortic aneurysm; DES, discrete event simulation; MASS, Multicentre Aneurysm Screening Study; NA, not available.

a Estimated for a sample size of 33,961 participants in the control group and 33,839 in the invited group, as in MASS.

### New Model Scenarios

**Figure 4:**
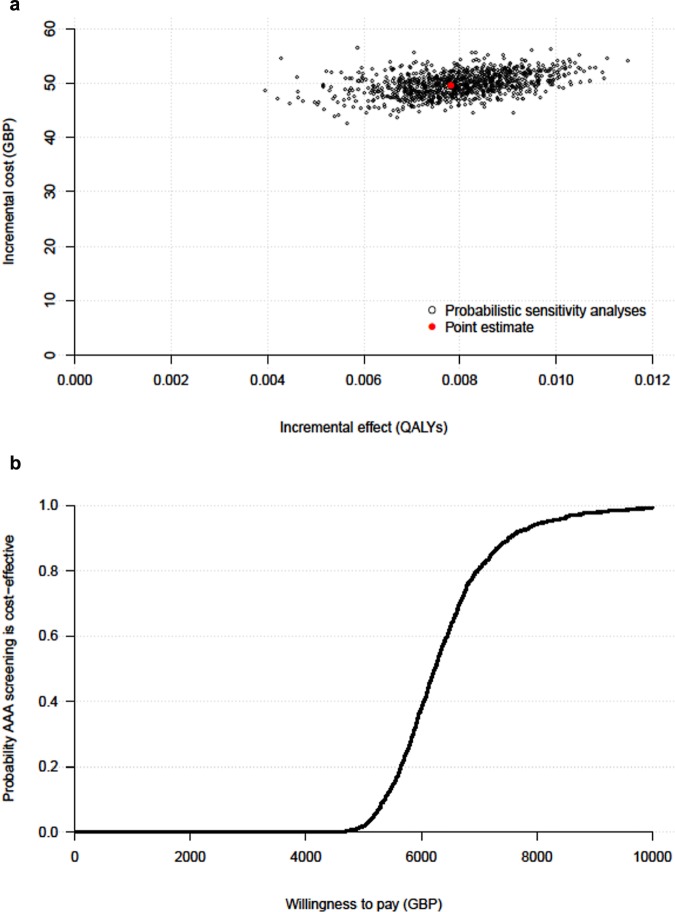
Long-term (30-y) cost-effectiveness of one-off invitation to AAA screening: (a) 1,000 probabilistic sensitivity analysis iterations (current NAAASP program), (b) cost-effectiveness acceptability curve. AAA, abdominal aortic aneurysm; NAAASP, National Health Service AAA screening programme.

The updated model after validation, using contemporaneous data sources, estimated a 30-y ICER of £6,352 (95%CI, £5,059 to £8,808) per QALY (Table 3). This compares to a 30-y ICER of £7,370 produced by the MM.^12^ The 1,000 iterations on the cost-effectiveness plane and cost-effectiveness acceptability curve shown in Figure 4 demonstrate that a one-off invitation to AAA screening and subsequent follow-up of identified AAAs is highly likely to be cost-effective, with no iterations outside the cost-effective region.

**Table 3:** Discrete Event Simulation: Long Term (30-y) Cost-effectiveness of One-off Invitation to AAA Screening for 65-y-old Men^a^

	DES Model
No invitation group	
Life-years	12.601
QALYs	9.681
Cost	£164
Invited group	
Life-years	12.611
QALYs	9.689
Cost	£213
Difference	
Life-years	0.01031
QALYs	0.00781
Cost	£50
ICER (QALYs)	£6,352
(95%CI)^b^	(£5,059 to £8,808)

DES, discrete event simulation; ICER, incremental cost-effectiveness ratio; QALY, quality-adjusted life year

a Life-years, QALYs, and costs discounted at 3.5% per y.

b Reported as uncertainty interval produced by 1,000 probabilistic sensitivity analysis iterations. Mean estimates from 1,000 PSA iterations for the difference in life-years, QALYs, costs, and the ICER were 0.01050, 0.00796, £50 and £6,388, respectively.

The estimated INMBs for both new scenarios were positive when compared with the existing screening program at a willingness-to-pay threshold of £20,000 per QALY (Table 4). The longer surveillance interval for the smallest AAAs (scenario 1) resulted in a small cost saving, as those with 3.0 to 3.9cm AAAs are screened less often than in the existing program. The longer surveillance interval was also associated with almost no change in QALYs. Extending the surveillance program to those with 2.5 to 2.9 cm aortic diameters (scenario 2) was associated with additional benefits but greater costs. However, the model suggests that rescanning these individuals at 5-y intervals could be cost-effective, with an INMB of £10 per individual invited to screening. The estimate of the INMB for scenario 1 was positive; however, the CI from the PSA included zero; the probability of it being cost-effective compared with the current strategy was 0.68. The INMB for scenario 2, with a CI that excludes zero, suggests that a surveillance for those with an aortic diameter between 2.5 and 2.9 cm is a cost-effective strategy as compared with the current strategy.

**Table 4:** Long-term (30-y) Cost-effectiveness. Scenario 1: Surveillance Intervals of 2 Y (3.0–3.9 cm AAAs), 1 Y (4.0–4.4 cm AAAs) and 3 Mo (4.5–5.4 cm AAAs). Scenario 2: Inclusion of Sub-aneurysmal (2.5–2.9 cm) AAAs in Screening Programme^a^

	Current Strategy	Scenario 1	Scenario 2
Mean incremental QALYs	0.00781	0.00781	0.00860
Mean incremental cost	£49.61	£48.54	£55.17
**Compared to current strategy:**			
Mean incremental QALYs	NA	0.00000	0.00080
Mean incremental cost	NA	-£1.07	£5.56
ICER (QALYs) (95%CI)^b^	NA	Dominant	£7,002 (4,615 to 12,233)
INMB^c^ (95%CI)^b^	NA	£0.99 (-2.03 to 3.35)	£10.33 (2.99 to 21.52)

ICER, incremental cost-effectiveness ratio; INMB, incremental net monetary benefit; QALY, quality-adjusted life year.

a Life-years, QALYs and costs discounted at 3.5% per y.

b At a willingness-to-pay of £20,000 per QALY.

c Reported as uncertainty interval produced by 1000 probabilistic sensitivity analysis iterations Mean estimates from 1000 PSA iterations for the difference in QALYs, costs, and the ICER for scenario 1 were 0.00000, £−1.08, and NA; and for scenario 2 were 0.00080, £5.58 and £7,233, respectively.

## Discussion

In assessing screening programs, modeling techniques are particularly relevant given that most of the costs are upfront, but benefits continue to accrue over time. The validated MM built by Kim and others^7^ demonstrated that AAA screening for men aged 65 y in the UK was likely to be cost-effective, but the model was inflexible when trying to address questions around configuration and optimization of screening. The creation of the DES has overcome these problems and the case of AAA screening highlights situations where DES may provide the most appropriate method to perform an economic evaluation, particularly when surveillance or rescreening is based on patient characteristics or risk markers (in this instance, AAA size). However, it cannot be asserted that the decision around the conceptual model should have been different at the onset of the research. The evolution of the decision problem has necessitated the re-conceptualization. Indeed, the MM served a valuable and timely purpose in showing the long-term cost-effectiveness of a one-off invitation to screening in the UK for men aged 65 y.

The DES built was based on the original MM, which itself was largely based on the MASS trial. This allowed the validation of the DES against observed MASS follow-up events and cost-effectiveness results. Overall, the DES validation process was similar to that for the MM, and no major changes were necessary to produce a comparison of key outputs. Given that some of the parameters in the model were not based on data directly observed in MASS (e.g., incidental detection rate), further calibration to fit MASS-observed data could have been undertaken to better replicate the number of events or the cost-effectiveness results based on trial data. This type of calibration was performed to produce a better fit using the MM.^24^ However, when trying to validate a model against data from a study in this way, there is a risk of creating a cyclical process. If too much information from the study is used, then the model output might match the data very well but the model may not predict well over a longer time horizon, rather like the issue of overfitting in statistical modeling. The differences between the DES and MM may be partly explained by the approach developed to handle aortic growth of those AAA <3.0 cm at first screen. The performance of the DES in the validation gives some confidence in using the model to extrapolate over a longer term.^12,23^

The general advantages of DES in health economic modeling have been extolled previously.^2,6,26–28^ They offer a decision modeler greater flexibility to adequately reflect clinical pathways, characterize baseline patient heterogeneity, allow event rates that change over time or depend on patient characteristics, and avoid the constraints of state transitions and fixed cycles imposed by the MM. In the case of AAA screening, there are 3 particular characteristics that mean that a DES is superior: firstly being able to define the size of a AAA as a continuous variable, which also allows measurement error in the ultrasound observations; secondly, allowing heterogeneity in the AAA growth rates between different individuals, with uncertainty easily characterised, something that is difficult to recreate in an aggregate discrete-state MM, even by varying the transition probabilities over time; and thirdly the ease with which time-varying surveillance intervals and other changes to the screening programme can be defined and evaluated. There are generally perceived trade-offs between the simplicity of an MM and the complexity of a DES, particularly related to model build time, potential data requirements, and model run times; the latter will always be a consideration. However, the advantages of DES models start to outweigh other factors as the complexity of the decision problem and modeled pathway increases, especially if structural modifications and further data analysis are necessary to deal with different scenarios.

The results of the scenarios presented here would be difficult to replicate in an MM, constrained by the state transition approach. The problem of modeling different surveillance intervals was encountered in previous work on AAA screening and required the re-programming of 6 different incarnations of the MM,^11^ each containing a different number of 3-mo tunnel states. For each surveillance interval that was different from the original screening strategy, tunnel states that allowed unobserved aortic growth and a related rupture rate in each cycle were necessary. Members of the cohort in these unobserved tunnel states were then able to move back into observed states at each rescan. It would have been desirable to explore more combinations of screening intervals and associated AAA size cut-offs. However, the structural changes that are a necessity in an MM would have made this a time-consuming process, thus limiting the number of analyses that could be considered. Conversely, the DES can easily assess any combination of surveillance intervals. To change the interval for patients with a 3.0 to 3.9 cm AAA from one to two years is trivial, because the DES is programmed to allow the input of any chosen partition of the aortic size range with an associated screening interval for each part. This is only possible because an individual’s AAA size is measured on a continuous scale. The problems of state transition are further demonstrated when trying to assess the cost-effectiveness of including sub-aneurysmal AAAs in a screening program. In the MM, a new AAA state (2.5 to 2.9 cm with 5-y surveillance intervals) would need to be incorporated, with extensive reprogramming. In the DES, all that is necessary is to insert “2.5” in the list of surveillance thresholds and “5” in the list of intervals. In addition, the DES parameters can also be easily made to depend on individual-level covariates (e.g., age-dependent mortality rates after surgery).

The DES can be used to assess the cost-effectiveness of other policy-relevant protocol changes, including the surgical threshold, the age at first screen, and recalling all those screened normal at first screen after a period of time. The model has been parametrized as part of the SWAN^29^ study and used to assess the likelihood of screening (with various protocols) women being cost-effective.

The restructuring of the model as a DES was, as might be expected, a relatively complex undertaking. Nevertheless, coding in a language such as R enables greater clarity and transparency compared to software designed for simulation modeling. However, the computational requirements of the DES were extensive, given the number of individuals needed to reduce sampling variation to an acceptable level and characterizing uncertainty through PSA. Run time was in the region of 24 h to run the model with 500,000 patients and 1,000 PSA iterations, even with parallelization and the use of a high-powered computer.

## Supplemental Material

DS_10.1177_0272989X17753380 – Supplemental material for Discrete Event Simulation for Decision Modeling in Health Care: Lessons from Abdominal Aortic Aneurysm Screening Click here for additional data file. Supplemental material, DS_10.1177_0272989X17753380 for Discrete Event Simulation for Decision Modeling in Health Care: Lessons from Abdominal Aortic Aneurysm Screening by Matthew J. Glover, Edmund Jones, Katya L. Masconi, Michael J. Sweeting, Simon G. Thompson, and SWAN Collaborators in Medical Decision Making
