# Supplementary material for: Discrete Event Simulation for Decision Modeling in Health Care: Lessons from Abdominal Aortic Aneurysm Screening
Source: Med Decis Making. 2018 Apr 2;38(4):439–51. doi: 10.1177/0272989X17753380 (PMC5950023; doi:10.1177/0272989X17753380)
Supplement: DS_10.1177_0272989X17753380 – Supplemental material for Discrete Event Simulation for Decision Modeling in Health Care: Lessons from Abdominal Aortic Aneurysm Screening [file DS_10.1177_0272989X17753380.pdf]

Supplementary Material

Modelling aortic growth

In the DES, the evolution of an individual's aortic diameter over time must be taken into account as it affects many aspects of the health economic model, namely: 1) when an individual can be diagnosed, 2) planned surveillance intervals, 3) when an intervention can be considered, 4) the risk of rupture, 5) the probability of receiving EVAR rather than open repair, and 6) the operative mortality risk. The evolution of the aortic diameter over time is therefore modelled using a continuous-time linear mixed model, which allows the underlying diameter and a measured diameter (using ultrasound or CT) to be determined at any time point. Let y_{ij} be the aortic diameter, as measured using ultrasound, of person i at time t_{ij}, $j = 1, \dots, n_i$; so y_{i0} is the baseline diameter as measured at screening. The linear mixed model is as follows:

$$\log(y_{ij}) = b_{0i} + b_{1i}t_{ij} + \epsilon_{ij}$$
$$= m_{ij} + \epsilon_{ij}$$
$$(b_{0i}, b_{1i})^T \sim N_2(\beta, G)$$

where

$$\epsilon_{ij} \sim N(0, \sigma_w^2)$$
$$\beta = \begin{pmatrix} \beta_0 \\ \beta_1 \end{pmatrix}$$
$$G = \begin{pmatrix} \sigma_0^2 & \rho\sigma_0\sigma_1 \\ \rho\sigma_0\sigma_1 & \sigma_1^2 \end{pmatrix}$$

Each person has two random effects: their own intercept (true baseline log diameter), b_{0i}, and their own slope (rate of growth), b_{1i}, measured on the log diameter scale. Correlation between an individual's underlying baseline log diameter and slope is allowed since b_{0i} and b_{1i} have a bivariate normal distribution with correlation parameter ρ. The parameters σ_0^2 and σ_1^2 determine the between-person variability of the intercepts and slopes, respectively, whilst σ_w^2 determines the amount of variability due to measurement error.

The linear mixed model is fitted using data from repeated ultrasound measurements of the aortic diameter from cohorts of AAA patients such as from MASS or RESCAN studies. These cohorts are restricted to include individuals whose initial diameters were in the range of 3.0 to 5.5cm. As a result, model extrapolation is used to infer true baseline diameters and growth rates for individuals outside of this range. Individuals with baseline aortic diameters <2.0cm are assumed to have no aortic growth (i.e. it is assumed that these individuals would never grow to be aneurysmal within their lifetimes).

Baseline diameter distribution and derived random-effects

The baseline diameter distribution is a particularly important aspect of the DES, because it determines how many persons have aneurysms at the time at which screening would be implemented and has a great effect on how many develop aneurysms in subsequent years. The full specification of the model is that y_{i0} follows a fixed baseline distribution, which we specify using external data sources (for example data on measured diameters from the first 700,000 men screened in NAAASP), and an individual's random effects b_{0i} and b_{1i} are then generated conditional on their observed baseline diameter. Following evaluation of the performance of the aortic growth model it was decided to use the following rules to generate an individual's random-effects:

1. If $y_{i0} \geq 3.0$ generate random-effects from the linear mixed model posterior distribution

Since estimated parameters from the linear mixed model are strictly relevant only to baseline diameters ≥3.0cm, then for individuals in this range, b_{0i} and b_{1i} are generated from their bivariate normal distribution conditional on the observed diameter, y_{i0}:

$$(b_i|y_{i0}) \sim N_2(\mu_b, \Sigma_b)$$

where

$$\mu_b = \beta + \begin{pmatrix} \sigma_0^2 \\ \rho\sigma_0\sigma_1 \end{pmatrix} \frac{\log(y_{i0}) - \beta_0}{\sigma_0^2 + \sigma_w^2}$$

$$\Sigma_b = \begin{pmatrix} \sigma_0^2 + \sigma_w^2 & \rho\sigma_0\sigma_1\sigma_w^2 \\ \rho\sigma_0\sigma_1\sigma_w^2 & \sigma_0^2\sigma_1^2(1-\rho^2) + \sigma_1^2\sigma_w^2 \end{pmatrix}$$

2. <u>If $y_{i0} < 3.0$ set an individual's true baseline diameter to their observed diameter</u>
   If the observed baseline diameter, y_{i0}, measures <3.0cm then we set $b_{0i} = \log(y_{i0})$. This avoids shrinkage of the true baseline diameter upwards towards the mean in the AAA cohort used to fit the linear mixed model (as estimated by $\exp(\beta_0)$).

3. <u>If $2.0 \leq y_{i0} < 3.0$ generate an individual's rate of growth from their posterior distribution conditional on b_{i0}</u>
   If $2.0 \leq y_{i0} < 3.0$ then b_{1i} is generated from a univariate normal distribution conditional on b_{0i}:

$$(b_{1i}|b_{0i}) \sim N(\mu_{b1}, \sigma_{b1}^2)$$

   where

$$\mu_{b1} = \beta_1 + \frac{\rho\sigma_1}{\sigma_0}(b_{0i} - \beta_0)$$

$$\sigma_{b1}^2 = (1-\rho^2)\sigma_1^2$$

4. <u>If $y_{i0} < 2.0$ set rate of growth to zero</u>
   This rule means that no individuals measured below 2.0cm at baseline will grow during their lifetime. In this range, it was felt that the model extrapolated estimates of growth could no longer be relied upon and instead it was assumed these individuals would never grow to be aneurysmal within their lifetime.

The effect of the extrapolation rules set out above was investigated in two ways; firstly by comparing the number of key events predicted by the DES in 4-years with observed MASS data (see Table 2), and secondly by comparing growth rates in individuals with measured baseline aortic diameters 2.6-2.9cm with data from the Gloucestershire surveillance study, where 1233 individuals with 2.6-2.9cm aortic diameters at screening were followed up over a 15-year time period. Results from this second validation exercise are presented in Supplementary Figure 1 and show a reasonable fit for the DES model.

Supplementary Figure 1. Progression of sub-aneurysmal (2.6-2.9cm) individuals to the diagnosis threshold of 3.0cm over a 30-year time horizon, and comparison with data from the Gloucestershire study.

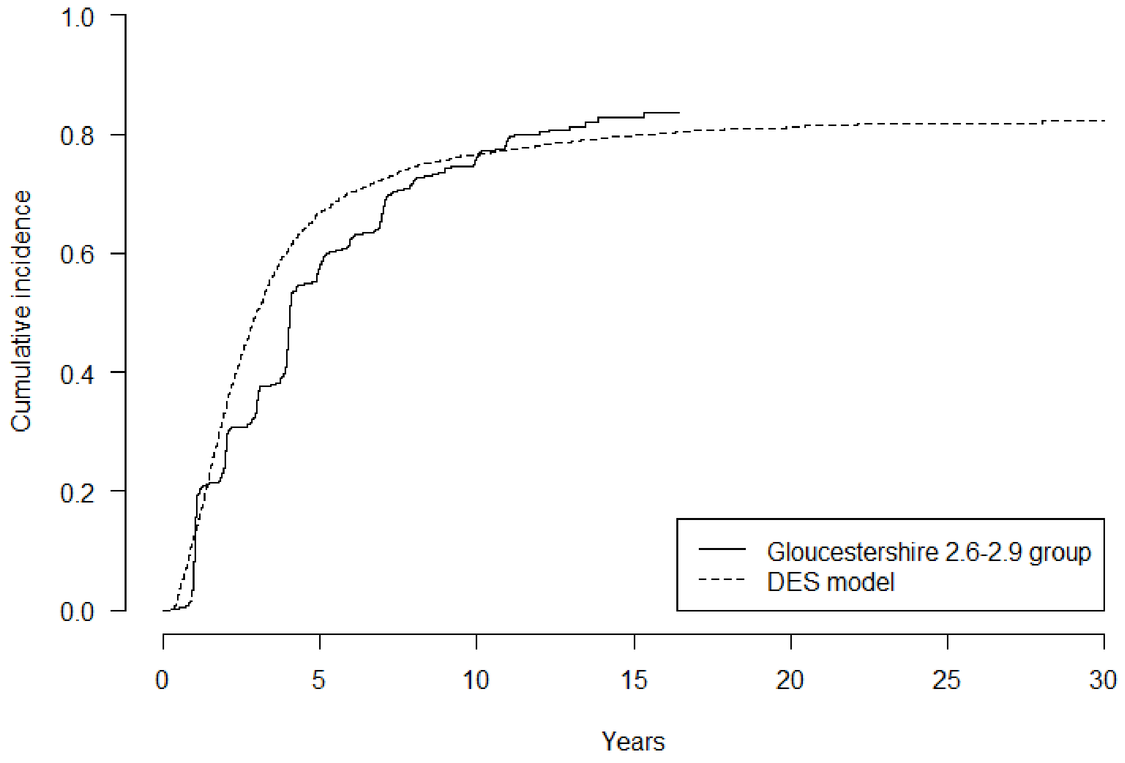

Supplementary Table 1. Parameters estimates used in the 4-year validation model and distributions used for a probabilistic sensitivity analysis

Parameter	Estimate	PSA Distribution	Source
Baseline diameter distribution	n/a	n/a	NAAASP[1]
Prevalence: p(≥ 3.0cm) at baseline	0.0497	Beta(1333, 25485)	MASS
Growth model parameters			
β_0 (log cm)	1.272	Multivariate normal[2]	MASS
β_1 (log cm/year)	0.058		
σ_0	0.176		
σ_1	0.036		
ρ	0.426		
σ_w	0.075		
Rupture model parameters			
γ	-16.263	Bivariate normal[3]	MASS
α	7.210		
**Probabilities**			
Require re-invitation	0.136	Beta(4602, 29237)	MASS
Attend screening	0.802	Beta(27147, 6682)	MASS
Non-visualization of aorta	0.0121	Beta(329, 26818)	MASS
Non-intervention (contraindicated)	0.135	Beta(46, 295)	MASS
Proportion receiving elective open vs EVAR	1	n/a	MASS
Elective open operative mortality	0.0373 [0.0992 [4]]	Beta(11, 284) [Beta(13, 118) [4]]	MASS
Elective EVAR operative mortality	n/a	n/a	n/a
Emergency surgery after rupture	0.441	Beta(90, 114)	MASS
Emergency open operative mortality	0.356	Beta(32, 58)	MASS
**Rates per person-year**			
Rate of post-operative AAA death following elective open	n/a	n/a	n/a
Rate of post-operative AAA death following elective open	n/a	n/a	n/a
Dropout from surveillance	0.082	Gamma(296, 0.00028)	MASS
Incidental detection	0.0755	$-4\log(1-X)$ where X~Beta(13.2, 691)	MASS
Non-AAA death after contraindication	0.234	Gamma(15, 0.01564)	MASS
Non-AAA death	Age-specific	n/a	MASS
**Costs (£)**			
Invitation to screen	1.31	n/a	MASS
Re-invitation	1.28	n/a	MASS
Screening ultrasound scan	19.08	n/a	MASS
Monitoring ultrasound scan	46.04	n/a	MASS
Consultation for elective surgery	309.88	n/a	MASS
Elective open surgery	6908.75	n/a	MASS
Emergency open surgery	11175.63	n/a	MASS
**Other**			
CT measurement difference μ_{CT} (cm)	0.2443	n/a	RESCAN
CT scan measurement difference σ_{CT}	0.190	n/a	Singh
Delay from large AAA scan to consultation (days)	71	n/a	MASS
Delay from consultation to elective surgery	59	n/a	MASS
Surveillance interval 3.0-4.4cm (months)	12	n/a	MASS
Surveillance interval 4.5-5.4cm (months)	3	n/a	MASS

Footnotes to Supplementary Table 1

[1] NAAASP distribution reweighted to give desired prevalence and the correct proportion of individuals with aortic size 3.0-4.4cm, 4.5-5.4cm and ≥5.5cm as in MASS

[2] PSA realisations generated from back transformation from a multivariate normal distribution with mean vector $(\beta_1, \beta_0, \log\sigma_1, \log\sigma_0, \operatorname{atanh}\rho, \log\sigma_w)$ and covariance matrix

$$\begin{pmatrix} 1.99\times10^{-6} & & & & & \\ 1.71\times10^{-6} & 3.01\times10^{-5} & & & & \\ 0 & 0 & 0.001714 & & & \\ 0 & 0 & -1.9\times10^{-5} & 0.000528 & & \\ 0 & 0 & 0.000483 & 4.84\times10^{-5} & 0.002588 & \\ 0 & 0 & 6.8\times10^{-5} & -1.4\times10^{-6} & 9.26\times10^{-6} & 8.09\times10^{-5} \end{pmatrix}$$

[3] PSA realisations generated from a bivariate normal distribution with mean vector (α, γ) and covariance matrix

$$\begin{pmatrix} 1.001459 & \\ -1.650784 & 2.758093 \end{pmatrix}$$

[4] via incidental detection

Supplementary Table 2 Parameters estimates used in the 30-year contemporary model and distributions used for a probabilistic sensitivity analysis

Parameter	Estimate	PSA distribution (30-year contemporary model)	Source
Baseline diameter distribution	n/a	n/a	NAAASP
Prevalence: p($\geq$ 3.0cm) at baseline	0.0134	n/a	NAAASP
Growth model parameters			
β_0 (log cm)	1.272	Multivariate normal[1]	MASS
β_1 (log cm/year)	0.058		
σ_0	0.176		
σ_1	0.036		
ρ	0.426		
σ_w	0.075		
Rupture model parameters			
γ	-16.263	Bivariate normal[2]	MASS
α	7.210		
**Probabilities**			
Require re-invitation	0.136	Beta(4602, 29237)	MASS
Attend screening	0.750	Beta(93170, 31022)	NAAASP
Non-visualization of aorta	0.0121	Beta(329, 26818)	MASS
Non-intervention (contraindicated)	0.125	Beta(69, 481)	MASS
Proportion receiving elective open vs EVAR	0.298	n/a	NVD
Elective open operative mortality	0.0411	Beta(24, 560)	EVAR-1
Elective EVAR operative mortality	0.0161	Beta(10, 612)	EVAR-1
Emergency surgery after rupture	0.368	Beta(193, 331)	MASS
Emergency open operative mortality	0.342	Beta(66, 127)	MASS
**Rates per person-year**			
Rate of post-operative AAA death following elective open	0.0007	Gamma(3, 0.00023)	EVAR-1
Rate of post-operative AAA death following elective EVAR	0.0077	Gamma(34, 0.00023)	EVAR-1
Dropout from surveillance	0.057	Gamma(330, 0.00017)	MASS
Incidental detection	0.0459	$-4\log(1-X)$ where X~Beta(19.56, 1695.95)	Glover
Non-AAA death after contraindication	0.247	Gamma(41, 0.006)	MASS
Non-AAA death	Age-specific	n/a	ONS
**Costs (£)**			
Invitation to screen	1.70	n/a	Glover
Re-invitation	1.70	n/a	Glover
Screening ultrasound scan	32.20	n/a	Glover
Monitoring ultrasound scan	68.00	n/a	Glover
Consultation for elective surgery	435.25	n/a	Glover
Elective open surgery	11532.69	n/a	Glover
Elective EVAR surgery	13345.66	n/a	Glover
Emergency open surgery	19984.75	n/a	Glover
**Other**			
CT scan measurement difference μ_{CT} (cm)	0.2443	n/a	RESCAN
CT scan measurement difference σ_{CT}	0.190	n/a	Singh
Delay from large AAA scan to consultation (days)	71	n/a	MASS
Delay from consultation to elective surgery	59	n/a	MASS
Surveillance interval 3.0-4.4cm (months)	12	n/a	MASS
Surveillance interval 4.5-5.4cm (months)	3	n/a	MASS

Footnotes to Supplementary Table 2

[1] PSA realisations generated from back transformation from a multivariate normal distribution with mean vector $(\beta_1, \beta_0, \log\sigma_1, \log\sigma_0, \operatorname{atanh}\rho, \log\sigma_w)$ and associated variance-covariance matrix

$$\begin{pmatrix} 1.99\times10^{-6} & & & & & \\ 1.71\times10^{-6} & 3.01\times10^{-5} & & & & \\ 0 & 0 & 0.001714 & & & \\ 0 & 0 & -1.9\times10^{-5} & 0.000528 & & \\ 0 & 0 & 0.000483 & 4.84\times10^{-5} & 0.002588 & \\ 0 & 0 & 6.8\times10^{-5} & -1.4\times10^{-6} & 9.26\times10^{-6} & 8.09\times10^{-5} \end{pmatrix}$$

[2] PSA realisations generated from a bivariate normal distribution with mean vector (α, γ) and associated variance-covariance matrix

$$\begin{pmatrix} 1.001459 & \\ -1.650784 & 2.758093 \end{pmatrix}$$
